# RNP-world: The ultimate essence of life is a ribonucleoprotein process

**DOI:** 10.1590/1678-4685-GMB-2022-0127

**Published:** 2022-09-23

**Authors:** Sávio Torres de Farias, Francisco Prosdocimi

**Affiliations:** 1 Universidade Federal da Paraíba, Centro de Ciências Exatas e da Natureza, Laboratório de Genética Evolutiva Paulo Leminski, João Pessoa, PB, Brazil.; 2 Network of Researchers on the Chemical Evolution of Life (NoRCEL), Leeds, UK.; 3 Universidade Federal do Rio de Janeiro, Instituto de Bioquímica Médica Leopoldo de Meis, Laboratório de Biologia Teórica e de Sistemas, Rio de Janeiro, RJ, Brazil.

**Keywords:** Ribosome, tRNAs, origin of life, gene origin, FUCA

## Abstract

The fundamental essence of life is based on process of interaction between nucleic acids and proteins. In a prebiotic world, amino acids, peptides, ions, and other metabolites acted in protobiotic routes at the same time on which RNAs performed catalysis and self-replication. Nevertheless, it was only when nucleic acids and peptides started to interact together in an organized process that life emerged. First, the ignition was sparked with the formation of a Peptidyl Transferase Center (PTC), possibly by concatenation of proto-tRNAs. This molecule that would become the catalytic site of ribosomes started a process of self-organization that gave origin to a protoorganism named FUCA, a ribonucleic ribosomal-like apparatus capable to polymerize amino acids. In that sense, we review hypotheses about the origin and early evolution of the genetic code. Next, populations of open biological systems named progenotes were capable of accumulating and exchanging genetic material, producing the first genomes. Progenotes then evolved in two paths: some presented their own ribosomes and others used available ribosomes in the medium to translate their encoded information. At some point, two different types of organisms emerged from populations of progenotes: the ribosome-encoding organisms (cells) and the capsid-encoding organisms (viruses).

## Introduction

The origin of life is one of the most important questions regarding the place of humans and living beings in the cosmos. Every human culture presents its own idea about how life was created in the old ages. Although this myriad of histories forms a rich cultural aspect of society, it is the origin of life as unraveled by modern science that is capable to integrate, unite and produce a trustworthy model that allow us to glimpse what actually happened in the past so that life could be originated and started to evolve. Therefore, it is under the scrutiny of scientific thought that those ancient questions can acquire a more elegant costume based both on observed facts and the accurate use of reason, logics and, of course, creativity.

The year 1953 can be considered a milestone in studies on the origin of biological systems, since both (i) the discovery of the DNA structure, by Watson and Crick, and (ii) the modern simulation of a prebiotic environment, by Stanley Miller, were published (Miller, 1953; Watson and Crick, 1953). Miller produced an ingenious apparatus capable to simulate the conditions of early Earth, in which he added the gases present at that time, being capable to observe the synthesis of biological molecules from simpler compounds. Previously, the hypotheses for the origin of life focused on the origin of organisms, and, since Miller, it was clear that the origin of life should have started from chemistry, focusing on the origin of the first biological molecules. With the advance of molecular biology along the late 1960s, the proposition that RNAs should have been the first informational molecule of biological systems came along (Woese, 1965; Crick, 1968; Orgel, 1968). About a decade later, in the early 1980s, a new step was taken with the proposition of a world dominated by those molecules of RNA. At that period, it was discovered that RNAs could present catalytic activity, a characteristic that was only known for proteins (Kruger et al., 1982; Guerrier-Takada et al., 1983). In 1986, this knowledge about the multiple functions performed by RNAs led the American biochemist and Nobel laureated Walter Gilbert to propose the RNA World model for the origin of biological systems (Gilbert, 1986). In the years that followed this proposal, several groups carried out experiments on the actual possibility of an RNA World, helping to advance the conceptual structuring of the model (Gilbert, 1986; Schwartz, 1995; Dworkin et al., 2003; Robertson and Joyce, 2012). In general terms, the idea of an RNA world suggested that early biological systems were composed of RNAs capable of performing the two main characteristics of this molecule: self-replication and catalysis. It would be in this context that the first metabolic routes were probably assembled, as well as the first system to store genetic information. The model suggested that the catalytic functions initially performed by RNAs would have been gradually replaced by peptides or proteins (Cech, 2009). Proteins are often accepted to be the more efficient catalysts because they are polymers formed by a chemically diverse set of 20 amino acids that allows them to present sophisticated tridimensional structures capable to interact much better with other molecules. Thus, biological systems would leave a world initially dominated by RNAs to establish a new world composed of RNAs and proteins, that is, a ribonucleoprotein (RNP) world.

Despite its great explanatory power and its wide acceptance in the scientific community, many criticisms have been made to the idea of an RNA world over the years. The main criticism to the model is related to the problem of producing the basic components for nucleotide formation in prebiotic contexts (Le Vay and Mutschler, 2019). Although there is still no consensus, it is possible that the existence of some prebiotic chemical refuges, containing abundance of certain atoms and molecules, facilitated the formation of nucleotides on early Earth. Mainly due to these reasons, new models have been proposed as alternative scenarios for the origin of biological systems. 

First, we must acknowledge that it is a consensus among the specialists that the RNA molecules were the first informational molecules of life and that DNA appeared later. On that matters, many researchers are suggesting a late appearance of DNA at quasi-cellular stage on which most of the biochemical pathways were already assembled (Forterre, 2005; Do Ó et al., 2020; Farias et al., 2021b; Di Giulio, 2021b). Two main facts strengthened the idea of the RNP world over the RNA world: (i) the abundance of amino acids in simulations of prebiotic environments and (ii) the understanding of the origin, importance, and evolution of ribosomes (Belousoff et al., 2010; Farias *et al.*, 2014; Petrov et al., 2015; Farias and José, 2020). 

In this review, we will address the main advances about the origin of biological systems from a ribonucleoprotein point of view. Such ideas range from the conceptual formulation of what life is to recent data explaining how the symbiotic, chemical interaction between RNAs and proteins were established on early Earth (Box 1).

**Box 1-:** Some authors and important ideas for modeling an RNP world.

Miller (1953) - Synthesized amino acids from simpler compounds. Palade (1955) - First observation of the ribosomal complex. Woese (1965), Crick (1968) and Orgel (1968) - Proposed that RNA could be the first informational molecule. Eigen and Schuster (1977a,b,c) - Proposed the hypercycles model for the initial organization of biological systems. Eigen and Winkler-Oswatitsch (1981) - Suggested that tRNAs acted as the first genes. Kruger et al. (1982), Guerrier-Takada and collaborators (1983) - Identified catalytic activity in RNA molecules. Bloch et al. (1984, 1989) - Found evidence for a common origin between tRNAs and rRNAs. Di Giulio (1997) - Proposes a model for the origin of coenzymes before the emergence of ribosomes. In his model, a ribonucleoprotein interaction is assumed for the origin of biological catalysis. Ban et al. (2000), Schluenzen et al. (2000) and Wimberly *et al.* (2000) - Unraveled the three-dimensional structure of the ribosome. Davidovich et al. (2009), Petrov et al. (2014) - Demonstrated that the peptidyl transferase center is the oldest portion of the ribosome. Farias et al. (2014), Root-Bernstein and Root-Bernstein (2015) - Demonstrated similarities between the peptidyl transferase center and tRNA molecules. Caetano-Anollés and Sun (2014) - Suggested that tRNA molecules are older than rRNA molecules. Keller et al. (2014) - Demonstrated the possibility of the glycolytic pathway and the pentose pathway to occur under prebiotic conditions without the presence of enzymes. Root-Bernstein and Root-Bernstein (2015) e Farias et al. (2016) - Found similarities between tRNAs and genes that encode basal processes in cells. Root-Bernstein and Root-Bernstein (2015) - Suggested that the ribosome functioned as a primitive genome. Lanier et al. (2017), Prosdocimi et al. (2021) - Suggested mutualism and chemical symbiosis, respectively, as scenarios for the emergence of biological systems. Farias et al. (2021) - Suggested a concept of life based on the processing of encoded information, with the origin of translation as a central point in the process of establishing biological systems. Bose et al. (2022) - Synthesized a primitive peptidyl transferase center capable of catalyzing random peptide bonds.

## Life: A conceptual problem

Before analyzing any model for the origin of life, we must consider how we should conceptualize this amazing phenomenon. Different concepts of life are related to different characteristics considered important during the transition from an abiotic world to a biotic world. Thus, different processes can be considered essential, bringing to different approaches. At this point, it is worth emphasizing the importance of relying on strong concepts that allow the identification of unique and exclusive characteristics, capable of distinguishing biological systems from other natural systems, either physical or chemical. In the specialized literature, more than one hundred different propositions of life concepts exist. Those concepts can be often separated and classified in three great groups based on their approach to biological systems as either (a) physical, (b) cellular, or (c) molecular concepts (Farias et al., 2021a). 

Concepts based on (a) the physical approaches often use some characteristics to define life, such as: (i) the decrease in entropy, and (ii) the distance from thermodynamic equilibrium. When observing biological systems, we can identify that they indeed present these characteristics indicated by the physical approach. However, we need to understand that these characteristics are not unique to biological systems and, therefore, cannot be used to draw a clear distinction between the living and the non-living. 

Regarding the concepts based on the (b) cellular approach, we can consider them as the most hegemonic nowadays, often describing general principles as (i) autonomy and (ii) the capacity for evolution. They indicate the need for a system to be compartmentalized and to present both informational molecules and metabolism (Ruiz-Mirazo et al., 2004). By autonomy, we understand the ability of an entity to maintain itself and reproduce independently. By the ability to evolve, we understand the possibility of genetic modification and the establishment of lineages. As a thought experiment, it is worth looking briefly at the role of viruses in this context. In the cellular approach, viruses have been excluded from the scope of life, since they do not present autonomy, even if they have evolutionary capacity. This exclusion of viruses from the scope of life, by itself, does not represent a conceptual problem. On the other hand, when analyzing the issue of autonomy, we can describe examples of organisms uncapable to establish lineages that are nevertheless alive according to the cellular approach (Prosdocimi and Farias, 2019). A classic example of this controversial issue are the infertile hybrids, such as the mule. Although mules are clearly alive, they are not targets of the evolutionary process because they are infertile and cannot pass genetic information ahead on their lineages. Thus, those pilar concepts that build the cellular approach to define life also fail to establish a set of unique and universal characteristics for living organisms (Farias *et al.*, 2021a). The viruses and the mules are clear counterexamples that demonstrate how autonomy and evolutionary capacity fail to be *bona fide* concepts to define life.

Finally, concepts related to (c) the molecular approach suggest that life should be understood as a self-sustaining chemical system that presents evolutionary capacity. Such as we observed with the cellular approach, there is a presumption of autonomy and evolutionary capacity. Thus, many authors argue that these two characteristics were already present in an RNA world (Joyce, 1994). RNAs indeed may have replicative and catalytic capacities and they could establish molecular lineages. For us, the main problem with concepts based on the molecular approach is that they assume that the emergence of replication and catalysis is sufficient to make a clear distinction between biological systems and other natural systems. However, both replication and catalysis can be found in chemical and/or physical systems. Thus, it is impossible to make a clear distinction between an abiotic world and a biotic world based only on replication and catalysis (Prosdocimi et al., 2018).

**Figure 1-:**
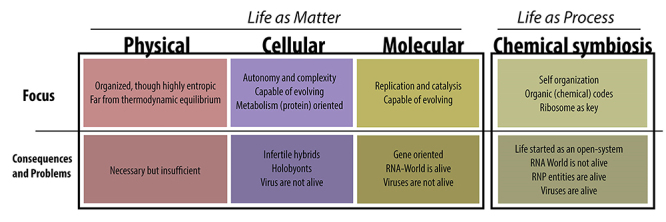
“Life as Matter” versus “Life as Process” scenarios: Central ideas and consequences for the main approaches to the concept of life.

When we observe the conceptual structure of those three different approaches presented above, we identify the use of typological characteristics as elements of distinction between the living and the non-living. Alternatively, it has been proposed that we should understand life as a process; and living beings should be seen as the materialization of this process (Farias et al., 2021a). Dupré and Nicholson (2018) argue that material entities should be understood as specific temporal stages based on stable processes. Thus, the stability and persistence of entities should be better understood as processes of spatial-temporal organization that presents causal and temporal relationship with other processes and entities. In line with these thoughts, Simons (2018) suggested that material entities should be understood as a sort of precipitation of the processes that maintain and stabilize them. Farias *et al.* (2021a), when analyzing the various processes that occur in biological systems, suggested that the processing of encoding information is unique and exclusive to living beings, being therefore a necessary and sufficient characteristic capable to distinguish the living from the non-living. In this way, these authors point to the emergence of the first encoded information system as the most important transitional feature between an abiotic world and a biotic world. Thus, it seems reasonable to focus our discussion and attention on the origin of the most basal coding system present in living organisms. This coding system is represented by the genetic code and therefore the most adequate approach to tackle the origin of life is understanding it from a ribonucleoprotein perspective related to the origin of the Translation system (Figure 1) (Prosdocimi and Farias, 2021). In this context, we should neither eliminate the idea of an RNA world nor deny the existence of protometabolic pathways at a certain stage in the history of Earth, but we should position these events as having occurred in a prebiotic era.

## Ribozymes, cofactors and the ancient relationship between nucleic acids and peptides

As we shall see, the idea that biological systems originated and evolved from a ribonucleoprotein core assumes that a chemical symbiosis between nucleic acids and proteins was necessary for the organization of life (Prosdocimi et al., 2021a). As we discussed earlier, the idea of an RNA world has been widely discussed in the scientific community due to the multiple functions of storing information and acting as catalysts found in RNA molecules (Gilbert, 1986). However, recent works indicated that the current ribozymes found in contemporary biological systems always contain a protein component assisting in their functions (Le Vay and Mutschler, 2019). This feature suggests that the association between proteins and nucleic acids is ancestral and that peptides probably aided in the RNA stability and functionality since long (Le Vay and Mutschler, 2019). 

This scenario invites us to consider the relevance of cofactors to the catalytic processes. Cofactors are often divided into two groups, the first formed by (i) inorganic ions, and the second formed by (ii) organic molecules, also known as coenzymes. The group of inorganic ion cofactors uses either molecules or single atoms that represent the first “catalytic agents” before the emergence of enzymes. In 2014, Keller and collaborators have shown that Fe^2+^ could be used to aid the synthesis of all intermediates of the glycolytic pathway and the pentose pathways under prebiotic conditions (Keller *et al.*, 2014). Second, most non-protein components of coenzymes are originated from nucleic acid molecules. The most significant example is Acetyl-CoA, a molecule often considered as pivotal to the cell metabolism, acting as intermediate in the biosynthesis of carbohydrates, proteins, and lipids. These facts corroborate the idea that a close relationship between nucleic acids and proteins happened at an earlier time than is currently assumed. Concerning this issue, the Italian researcher Massimo Di Giulio presented a model for the emergence of the coenzyme-enzyme interaction in 1997. According to this recognized researcher, the nucleotidic component of the coenzymes was already associated with amino acids. Only from this initial relationship a progression in the synthesis of both the nucleotide component and the protein component was possible. Then, at the end of this molecular evolutionary process, the nucleotide would lose its interaction with the protein and leave only the coenzyme component to assist the catalytic function (Di Giulio, 1997). Curiously, Di Giulio suggests that RNAs and peptides were probably linked by covalent bonds at that time, a binding only observed today in peptidyl tRNAs. In a recent article, the author suggested that this interaction between peptides and tRNAs were probably the trigger that initiated the process of encoding biological information, followed by the formation of proto-mRNAs and the organization of the genetic code (Di Giulio, 2021a). Under those assumptions, it is even possible to consider the existence of protein synthesis without the presence of ribosomes. It is interesting to note that many coenzymes are derived from nucleotides, some of which are made up of either ribonucleosides or entire ribonucleotides. These components may have helped in the complexification of peptides into small proteins. Thus, it has been suggested that the domains of globular proteins were selected for their ability to bind to these types of cofactors. This suggestion comes from the fact that their most ancestral protein domains were shown to be exactly the ones capable to bind to the nucleotide derived cofactors (Fried et al., 2022). The model presented by both Di Giulio (1997, 2021b) and Fried *et al.* (2022) is compatible with the data observed in modern organisms. This fact implies that the ribonucleoprotein world cannot be understood as a transitory stage, but as the essence of life itself, that comes since the primordial organization of life and endured until today, as observed in contemporary organisms. Therefore, we saw that ribozymes cannot function well without associated amino acids or peptides and that many proteins are aided by the existence of nucleotide-derived cofactors. Both facts are evidence of the intimate relationship between these key macromolecules that constitutes the ultimate essence of life. 

## Chemical symbiosis theory: From FUCA to LUCA

In 2021, Prosdocimi and colleagues suggested the theory of chemical symbiosis based on a Margulian view of the biotic world (Prosdocimi *et al.*, 2021a). In their model, it is proposed that biological systems were initially established through a collaboration between peptides and nucleic acids, where neither of the two macromolecules would have been able to produce life without the aid of the other. Thus, the authors identified the origin of the ribosome, as well as the origin of the whole translation system, as the founding event of life. The process of establishing a symbiosis relationship between nucleic acids and peptides led to the emergence of a “First Universal Common Ancestor” (FUCA) (Prosdocimi *et al.*, 2019). In this context, the emergence of FUCA is directly linked to the first steps that led to the formation of the genetic code, and its maturation was achieved with the completion of the first chemically encoded information system. 

**Figure 2 -:**
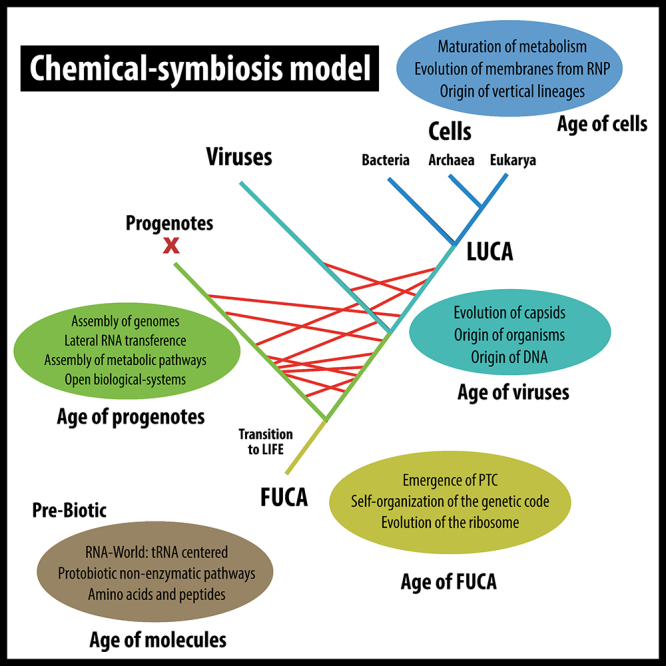
The chemical symbiosis model: a RiboNucleoProtein scenario for the origin of biological systems. The ages and their most conspicuous features are highlighted in the balloons. Previously to the origin of cells, reticulated evolution operated by lateral transference of genetic material (red lines).

The term FUCA describes a very early period in the organization of biological systems and complements the concept of LUCA, which has its meaning associated with the emergence of the first biological systems that were cellular. FUCA is, therefore, the earliest ancestor of LUCA. Thus, it is suggested that the initial organization of biological systems would have taken place in a semi-open environment, with intense exchange of information between the subsystems. There, in that primitive open environment, the translation system would have emerged and evolved with the origin of the first metabolic pathways (Prosdocimi and Farias, 2021). At that point in the evolution of life on Earth, organisms as we know them today were absent, but there existed molecular biological systems capable to process and metabolize information encoded in nucleic acids. We call this era as “the age of progenotes”, when recycling a term originally proposed by Carl Woese to describe this moment before the emergence of viruses and cells, that is, before the emergence of organisms as individuals (Woese, 1998). Thus, it is proposed that we should divide living organisms into two groups that could be considered as (i) ribosome-encoding entities, and (ii) capsid-encoding entities (Forterre and Prangishvili, 2009). This way of looking at organisms is in accordance to the model of a biological world that was open at its origins. In that period, the ribosome was at the center and was responsible to process the information contained in the different subsystems. As there was no compartmentalization yet, all open systems could access free ribosomes available in the medium (Prosdocimi *et al.*, 2021b). The maturation of these open biological systems or progenotes would then have led to the compartmentalization of subsystems. Thus, those systems that encapsulated themselves together with ribosomes established lineages we know now as cellular. On the other hand, those systems which encapsulated without ribosomes would establish viral lineages (Figure 2). Under the current proposal, as we shall see, the membrane will evolve from proteins capable to bind lipids (Sojo, 2019). Farias *et al.* (2019) suggested that organisms should be understood as stable life strategies and that the relationship between viruses and cells should be understood as a relic from the era of progenotes. In this sense, in their primordial relationship, viruses would not need cells to replicate, but ribosomes, which were necessary to process their information encoded in nucleic acids into protein (Prosdocimi and Farias, 2021). Encapsulation in cellular and viral systems would have inaugurated a new era in biological systems, the era of organisms and would have finally given rise to what we have come to know as the Last Universal Common Ancestor (LUCA), the progenitor line of all cellular diversity found today (Farias *et al.*, 2021a).

## The origin of ribosomes and the organization of FUCA

Coming back along the argumentation for a while, we must make clear that the translation system should therefore be considered as the heart of biological systems. This is important because, along this process, an organized conversation has taken place between the information contained in the nucleic acids and the manufacture of peptides and proteins. The establishment of this informational correspondence was carried out by the genetic code, which is considered the first truly biological code to emerge (Barbieri, 2018; Prosdocimi and Farias, 2021). The most important evidence for the RNP world is the understanding that the heart of the translation system is the ribosome, *i. e.*, a macromolecular complex composed of RNAs and proteins. 

The first observations of the ribosomal complex were made in the 1950s (Palade, 1955). However, it was only in the early 2000s that its structure was elucidated at the molecular level (Ban et al., 2000; Schluenzen et al., 2000; Wimberly *et al.*, 2000). These studies demonstrated that, despite the ribosomes being constituted by RNAs and proteins, in their catalytic site there is no protein enzyme, but rather, the catalytic reaction is carried out by a ribozyme. The characterization of the catalytic activity of ribosomes as an activity carried out by a ribozyme gave extra support and allowed this molecule to be repositioned with more certainty at the origin of the biological systems (Belousoff et al., 2010; Fox, 2010; Tirumalai et al., 2021). This fact also paved a new way to consider seriously that the origin of life would have happened in a ribonucleoprotein world.

Some studies that tried to reconstruct the genome of the last common ancestor have shown that the components of the translation system are the most conserved and abundant (Koonin, 2003; Delaye et al., 2005; Ouzounis et al., 2006; Weiss et al., 2016), supporting the antiquity of this process in biological systems. Comparative studies that analyzed the structural evolution of the larger ribosomal subunit demonstrated the conservation of its catalytic structure (Davidovich et al., 2009; Petrov et al., 2014; Farias et al., 2014; Bowman et al., 2020). In these studies, it has been suggested that the peptidyl transferase (PTC) center was probably the first part of the ribosome to self-organize. Farias *et al.* (2014) and Root-Bernstein and Root-Bernstein (2015), when performing comparative analyses between tRNA and rRNA molecules, observed that there were similarities between these molecules and suggested that the PTC and tRNAs have a common origin. Caetano-Anollés and Caetano-Anollés (2016) analyzed the data from Root-Bernstein and Root-Bernstein (2015) and proposed a chronology between the assembly of the genetic code and the structuring of ribosomes. In their study, they demonstrate that cognate tRNAs for the first amino acids are homologous to older regions of the ribosome. Historical data indicated a similarity between tRNAs and rRNAs molecules since the 1980s, but the lack of information in databases limited these studies (Bloch et al., 1984, 1989). Farias *et al.* (2017) modeled the three-dimensional structure of the ancestral PTC from ancestral tRNA sequences and observed a structural similarity of 92% when compared to the PTC of *Thermus thermophilus*. Using molecular docking experiments, it has been demonstrated that the ancestral PTC already had the ability to bind to tRNA molecules in a similar way to what we observe in modern organisms. This study also confirmed that the site where the nascent peptides leave the ribosome towards the cytosol, named the “exit tunnel”, already existed in the early ribosomal complex. Prosdocimi et al. (2020) analyzed the identity elements of PTC and identified the participation of tRNAs in the secondary structuring of this molecule. In this sense, it was suggested that the PTC initially worked by synthesizing peptides quasi-randomly, without the need for a genetic code, though respecting the availability of amino acids in the medium. Davidovich *et al*. (2009) analyzed the structure of PTC and identified two symmetrical structures together with a structure similar to tRNAs, suggesting that the PTC evolved by duplication. Bose et al. (2022) synthesized proto-rRNAs (proto-PTC) based on *T. thermophilus* rRNA and confirmed the catalytic ability to form peptide bonds randomly. In this context, it is interesting to notice that the proto-rRNAs that showed catalytic activity were similar to the same portions that showed similarities with tRNAs in the studies by Farias *et al.* (2014) and Root-Bernstein and Root-Bernstein (2015). Based on the data presented above, a common origin has been proposed between tRNA molecules and the catalytic site of the ribosome, which may have been formed by the concatenation of proto-tRNAs (Farias *et al.*, 2014; Prosdocimi *et al.*, 2020).

Farias et al. (2021b) performed a comparative analysis between ancestral tRNAs and the 16S ribosomal molecule and found similarities between these molecules at the 3’ Upper domain, a similar result to that found earlier by Root-Bernstein and Root-Bernstein (2015). Harish and Caetano-Anollés (2012) suggested that the smaller subunit of the ribosome is the oldest portion of this molecule. Farias *et al.* (2021b) modeled the three-dimensional structure of the ancestral 16S rRNA and performed a comparative analysis with the homologous portion of *T. thermophilus*, *Escherichia coli* and *Mycobacterium smegmatis*, observing a similarity in structure at the level of 94%, 90% and 86%, respectively. It is interesting to note that the “decoding site” is found at the 16S rRNA molecule, at the position on which the interaction with the mRNA occurs. Thus, we can see that the 3’ Upper domain is part of the decoding site by structuring both the peptidyl site (P) and the aminoacyl site (A) along the translation process. It is known that the universally conserved G530, A1492 and A1493 of 16S ribosomal RNA, critical for tRNA binding in the A site, actively monitor cognate tRNA, and that recognition of the correct codon-anticodon duplex induces an overall ribosome conformational change (domain closure) (Demeshkina et al., 2012). Demongeot and Seligmann (2019) analyzed the secondary structure of tRNAs and 16S rRNAs and suggested that the structure observed in 16S rRNA derived probably from ancestral tRNAs. All these data together have reinforced a ribonucleoprotein scenario for the beginning of biological systems and positioning of tRNAs as central molecules in the initial organization of biological systems.

## The origin of the first genes and the structuring of metabolism

The origin of the biological information system is a crucial point in understanding the formation of a ribonucleoprotein world (Prosdocimi and Farias, 2019). Evidently, when we talk about the origin of biological information, we must keep in mind the formation of the correspondence system between nucleic acids and peptides, that is, the genetic code. The understanding about the origin and organization of the genetic code is still a major challenge for researchers around the world, especially when we seek to understand the steps for the formation of this decoding information structure (Kuhn and Waser, 1994; Hartman, 1995; Di Giulio, 2005; Farias *et al.*, 2007; Guimarães et al., 2008; Rodin and Rodin, 2008; Trifonov, 2009; Guimarães, 2014; Koonin and Novozhilov, 2017; Yarus, 2021). Although we still do not have a definitive model that satisfactorily explains how the formation of the genetic code occurred, some general models are guidelines for further investigations. Among these models, it is worth mentioning (a) the stereochemical model and (b) the model of co-evolution between metabolic pathways and the genetic code.

In (a) the stereochemical model, it is suggested that the establishment of the correlation between amino acids and their codons has taken place by chemical affinity. In this sense, we can observe that there is a correlation of hydropathy between the charged amino acid in its cognate tRNA and the main dinucleotide of the anticodon (Farias et al., 2007; Guimarães, 2014). An interesting fact to notice is that, in modern organisms, the acceptor arm of the tRNA and the anticodon loop is separated by about 75 Angstroms and interact with distinct portions of the aminoacyl-tRNA synthetases, the enzymes responsible for charging activated amino acids to their cognate tRNAs. Some studies have also demonstrated that tRNAs having only the acceptor arm can be specifically linked by their corresponding aminoacyl tRNA synthetases. This observation led to the proposition that the genetic code was preceded by a sort of “operational code” and that it was only later that the tRNA molecule matured and the anticodon arm was incorporated (Schimmel et al., 1993; Hipps et al., 1995). Interestingly, Shimizu (1995) carried out experiments with only the anticodon loop and found that this portion also has the ability to bind its amino acid in a specific way. A solution for this apparent impasse was provided by Dantas et al. (2021) in a study on which ancestral sequences and structures for the class I aminoacyl tRNA synthetase were reconstructed. The researchers conducted molecular docking experiments between (i) the acceptor arm and (i’) the anticodon loop with the ancestral structures of the cognate aminoacyl tRNA synthetases. The results suggested that, at the origin of biological systems, these two portions of the tRNA would act independently and would bind in the same region of the enzyme: the modern catalytic site of the enzyme. Thus, it was only with the maturation of the aminoacyl tRNA synthetases that these portions of the tRNA would have modified their points of interaction and acquired the structure that we can observe today in modern organisms. These data also explain why we observed a hydropathic correlation between amino acids and the major dinucleotide of their cognate anticodons (Guimarães *et al.*, 2008; Dantas *et al.*, 2021).

In the (b) coevolution model, it is suggested that the amino acid synthesis pathways and the establishment of the genetic code co-evolved, and it is assumed that the amino acids were incorporated into the coding system as their synthesis pathways were established. Guimarães (2017) suggests that the amino acid synthesis pathways and the entire basal metabolism have been structured from the Glycine and Serine synthesis pathways. These pathways, after being structured, would have provided compounds for the formation of glycolysis and gluconeogenesis, which enabled the development of pathways for the synthesis of other amino acids later incorporated into the genetic code. Evidently, both the stereochemical model and the coevolution model cannot be seen as mutually exclusive since they may represent different phases of the same process.

Although it is extremely important to understand how the genetic code was structured, this information alone cannot explain where the first sequences (that had the potential to produce peptides when read by the genetic code) came from. In the quest to understand how the first genes appeared, a historical work by Eigen and Winkler-Oswatitsch (1981) suggested, based on the characteristics presented by tRNAs, that these molecules could have given rise to the first genes during the initial organization of biological systems. Based on these ideas, Farias et al. (2016a) reconstructed the ancestral sequences for the tRNAs and built concatamers consisting of three ancestral tRNAs in every possible combination. Such concatamers were aligned against the entire modern protein database (nr). In their results, it was possible to observe that, when translated, the ancestral tRNA concatamers showed similarities with several modern proteins. Many of those proteins were shown to operate in pathways considered to be essential and/or basal, such as: glycolysis, pentose pathway, translation proteins, amino acid synthesis, nucleotide synthesis, and lipid synthesis (Farias *et al.*, 2016a). Among the various proteins that had similarity to ancestral tRNAs was an RNA-dependent RNA polymerase capable of replicating RNA molecules (Farias *et al.*, 2017). Root-Bernstein and Root-Bernstein (2015, 2016, 2019), when analyzing the similarity between tRNA molecules and modern genes, obtained similar results to those found by Farias *et al.* (2016a). In their studies, these authors propose that the ribosome may have acted as a primitive genome, in the early days of the biological system (Root-Bernstein and Root-Bernstein, 2015; 2016, 2019). Faure and Barthélémy (2018) analyzed mitochondrial genomes and identified that tRNA genes had a high frequency of translation start and stop signals. Additionally, Farias *et al.* (2017) reconstructed the three-dimensional structure of the protein sequence derived from the junction of tRNAs that had similarity to RNA-dependent RNA polymerase. The researchers observed that this part of the protein had structural similarity to the catalytic domain of modern structures and the ability to bind magnesium. In this study, a structural distance tree was constructed, and the results indicated that ancestral structures were more similar to proteins found in ancestral families of viruses. Do Ó et al. (2020) reconstructed the structure of translated proteins from ancestral tRNAs and observed similarities to proteins of the glycolytic pathway. The results presented by them indicated that the catalytic site must have been the first part to be structured. However, searches for possible ligands indicated that those ancestral peptides should not act, in principle, as catalysts but rather as RNA stabilizers. Thus, they would have been co-opted later into the metabolic functions observed in modern organisms, in a clear example of what Stephen Jay Gould called exaptation (Gould and Vrba, 1982).

Furthermore, several studies of protobiotic chemistry have shown that pathways such as glycolysis, the pentose pathway, the citric acid cycle and others could occur in a prebiotic environment without the presence of biological catalysts (Keller et al., 2014; Muchowska et al., 2019a,b). These data suggest that, during the process of formation of biological systems, proteins replaced functions previously occupied by chemical catalysts, such as Iron (Fe^2+^) and other metallic ions (Keller *et al.*, 2014). In that work, Keller and collaborators present evidence that iron and other ions could act as catalysts previously to the existence of enzymes. In this sense, it is worth noticing that the pathways of amino acid synthesis derive either from the glycolytic pathway or from the citric acid cycle in modern organisms. Thus, the entry of biological catalysts in these processes must have increased the efficiency of the process and contributed to the increase in amino acid synthesis. This increase would supply the maturing translation system. Based on evidence of similarity between ancestral tRNAs, rRNAs and mRNAs, Farias et al. (2016b) proposed the model called “tRNA core hypothesis”. In this model, it is suggested that tRNAs or proto-tRNAs would function as organizers of the primitive translation system, giving rise to the catalytic site of the ribosome and the decoding site. They must also have functioned as the first informational molecules. In this way, the translation system clearly functioned as an attractor for the emergence and evolution of the first biological systems. In its origin, the entire biological system was selected to supply the process of translation of biological information and provide the molecules necessary for this process to occur optimally. These data reinforce the idea proposed by Eigen and Winkler-Oswatitsch (1981) about the relationship between tRNAs and the origin of the first genes, as well as the notion of a ribonucleoprotein world for the origin of biological systems. Thus, the translation process, together with all its main elements, was the protagonist at the initial organization of biological systems.

## Final Considerations

In science, explanatory models are built from the set of available evidence on a particular field of knowledge. Evidently, increasing knowledge about a given phenomenon can either strengthen or weaken an existing model or else allow the emergence of alternative models. After the discovery of the structure of the DNA molecule and the deepening of knowledge about the molecular functioning of organisms, the RNA molecule gained prominence in explanatory models about the origin of biological systems. This emphasis was not given by chance, but rather because of a solid body of evidence that indicated its importance at this early stage of life (Woese, 1965; Crick, 1968; Orgel, 1968; Kruger et al., 1982; Guerrier-Takada et al., 1983). In this context, the idea of an RNA world to represent the beginning of biological systems has taken shape and has been strengthened in the last 30 years (Gilbert, 1986; Schwartz, 1995; Dworkin et al., 2003; Robertson and Joyce, 2012). However, from the beginning of the 2000s, with the elucidation of the ribosomal structure, together with new conceptual views on the biological systems have been strengthening a ribonucleoprotein view for the emergence of biological systems. These new models do not rule out the RNA molecule as the first informational molecule but place these molecules working in symbiosis with protein molecules since the origin of what we know as life. 

Here, we review the proposal that the transition from an abiotic world to a biotic world took place through the establishment of the most basal language used by biological systems. Our contributions, amongst many others, suggest that the capacity to communicate the information contained in nucleic acids to the information contained in proteins, that is, the genetic code, was the pivotal event that allowed life to emerge (Farias et al., 2021a). The emergence of the genetic code allowed the emergence of a first proto-organism named FUCA, which was initially structured as a semi-open system, on which the primitive translation system acted as an attractor for structuring the entire biological system (Prosdocimi et al., 2019). After a first stage of genetic code maturation, the whole translation system has been established. Then, the first metabolic pathways were assembled by collaboration between the open-systems called progenotes. These systems functioned initially in a semi-open way accessing free ribosomes in the medium and translating their encoded information. This further allowed encapsulation of proteins that fit together and then inaugurated the era of organisms, possibly starting with virus-like structures. The age of organisms allowed the establishment of individual lineages and, in this way, allowed the emergence of the basic structures of life as we know it, namely: viruses, bacteria, and archaea. Although several aspects still need to be unveiled about the origin and initial evolution of biological systems, the current knowledge about the molecular functioning of these systems has been opening new perspectives and provided more and more the elaboration of complex and complete models on this fascinating question approached by virtually every human culture. However, although we have come a long way in recent years, the road is still long and full of surprises. Only with the perseverance in the study of nature will we be able to either validate current models or design new scenarios. 
